# Human Umbilical Cord Mesenchymal Stem Cells Alleviate LPS-Induced Acute Lung Injury in Mice: Association with TLR4/MyD88/NF-κB Pathway Suppression

**DOI:** 10.3390/biomedicines14071632

**Published:** 2026-07-20

**Authors:** Mingyou Yu, Ziyi Zhang, Ying Hu, Jinhui Zhang, Panpan Lu, Jingyu Luo, Jianwei Xu

**Affiliations:** 1 Center for Tissue Engineering and Stem Cell Research, Translation Medicine Research Center, Guizhou Biomanufacturing Laboratory, Guizhou Medical University, Gui’an New Area, Guiyang 561113, China; yumingyou2026@163.com (M.Y.); zhangziyi1774323@163.com (Z.Z.); 2023110010118@stu.gmc.edu.cn (J.Z.); 13984412490@163.com (P.L.); 2 School of Pharmacy, Guizhou Medical University, Guiyang 550031, China; 3 School of Basic Medicine, Guizhou Medical University, Guiyang 550031, China; hy1774323@163.com (Y.H.); gzykdz16854@163.com (J.L.)

**Keywords:** acute lung injury, human umbilical cord mesenchymal stem cells, TLR4/MyD88/NF-κB pathway, inhibition of inflammation

## Abstract

**Objective:** In a lipopolysaccharide (LPS)-induced acute lung injury (ALI) mouse model, the present study sought to assess the therapeutic efficacy of human umbilical cord-derived mesenchymal stem cells (hUC-MSCs) and characterize their anti-inflammatory mechanistic basis. **Methods:** Forty mice were randomly divided into four groups: control, LPS model, LPS + DEX (positive control), and LPS + hUC-MSCs. Except for the control group, mice received intratracheal instillation of LPS to establish ALI. One hour after LPS administration, animals in the hUC-MSC group were intravenously infused with hUC-MSCs. The positive control group was given an intraperitoneal injection of DEX for 3 consecutive days, starting at 24 h after modeling. On day 4 after cell transplantation or at 24 h after the completion of DEX injection, lung function indicators were detected. Bronchoalveolar lavage fluid (BALF), serum, and lung tissues were subsequently obtained for evaluation of inflammatory cell infiltration, histopathological injury, lung wet-to-dry (W/D) ratio, and cytokine levels. Additionally, the localization of transplanted hUC-MSCs in lungs was examined, and the mRNA and protein expression levels of *TLR4*, *MyD88*, and *NF-κB p65* were quantified. **Results:** LPS exposure markedly impaired pulmonary function and induced robust inflammatory responses, evidenced by elevated levels of pro-inflammatory cytokines, increased inflammatory cell counts in BALF and serum, and extensive histological lung damage. Moreover, hUC-MSC injection improved lung function, decreased inflammatory cytokine production and alleviated pulmonary edema, while inhibiting the TLR4/MyD88/NF-κB pathway at transcriptional and protein levels. **Conclusions:** Intravenous hUC-MSC administration alleviates LPS-induced ALI in mice, an effect associated with suppression of the TLR4/MyD88/NF-κB cascade. These results indicate that this signaling cascade partially mediates the observed anti-inflammatory effects.

## 1. Introduction

Acute lung injury (ALI) is a pronounced respiratory condition marked by extensive pulmonary inflammation, which remains linked to substantial fatality rates [1,2]. Among the various causes of ALI, sepsis secondary to bacterial pneumonia is the most frequently encountered [3]. During bacterial infection, innate immune activation promotes the accumulation and priming of alveolar macrophages and neutrophils in lung tissue. These inflammatory cells produce large amounts of cytokines, comprising tumor necrosis factor-alpha (TNF-α) and interleukin-1β (IL-1β), amplifying the inflammatory cascade and damaging pulmonary epithelial and endothelial barriers [4]. Persistent and uncontrolled inflammation may eventually develop into acute respiratory distress syndrome (ARDS), resulting in fatal pulmonary dysfunction [5]. Therefore, the timely termination of inflammation is essential for reducing lung injury as well as reconstructing homeostasis [6]. Nevertheless, current management of ALI is still largely limited to supportive care, and specific pharmacological interventions remain insufficient.

Mesenchymal stem cells (MSCs) have attracted growing interest as a promising therapeutic strategy for inflammatory lung diseases [7]. MSCs can be obtained from several tissues, such as bone marrow and umbilical cord tissue [8]. Previous studies have reported that bone marrow-derived MSCs (BM-MSCs) exert protective effects in ALI, bacterial pneumonia, and sepsis [9,10]. However, clinical application of BM-MSCs is limited by the invasiveness of bone marrow harvest and the reduced proliferation and differentiation capacity in aged donors [11]. Compared with BM-MSCs, human umbilical cord-derived MSCs (hUC-MSCs) possess several strengths, including noninvasive collection, greater proliferative capacity, and relatively low immunogenicity [12,13]. These characteristics make hUC-MSCs a novel intervention for lung injury therapy [14,15]. Previous studies have shown that intratracheal administration of hUC-MSCs suppresses macrophage-mediated inflammatory responses, enhances Interleukin-10 expression and improves survival in lipopolysaccharide (LPS)-induced ALI mice [16]. In addition, microRNA-377-3p secreted from hUC-MSC exosomes also alleviates ALI in a mouse model [17]. Nonetheless, the underlying molecular mechanisms by which MSCs attenuate ALI have yet to be clarified.

Studies have exhibited that MSCs are able to attenuate LPS-induced ALI by suppressing the nuclear factor-κB (NF-κB) and Hedgehog pathways [18]. As a key downstream component of Toll-like receptor 4 (*TLR4*) signaling, NF-κB activates following myeloid differentiation factor 88 (*MyD88*) accumulation by *TLR4*, then translocates into the nucleus to trigger the production of pro-inflammatory mediators, thereby contributing to the inflammatory cascade [19]. However, whether the therapeutic benefits of MSC transplantation in LPS-induced ALI are mediated via the TLR4/MyD88/NF-κB signaling pathway remains unclear.

Here, we employed an LPS-induced ALI model, which is widely used to establish murine models of acute lung injury [20,21,22], to confirm that hUC-MSC transplantation markedly alleviated ALI by reducing pulmonary inflammation, thereby improving pulmonary function. Mechanistically, these therapeutic benefits were induced by inhibition of the TLR4/MyD88/NF-κB pathway as well as expression of cytokines. In addition, transplanted hUC-MSCs were observed to localize within injured lung tissue following administration. Collectively, our findings reinforce the therapeutic prospects of hUC-MSC-based cell intervention for ALI.

## 2. Materials and Methods

### 2.1. Drugs and Reagents

We acquired the following drugs and reagents for this study: lipopolysaccharides (LPS, Sigma, St. Louis, MO, USA; batch number: 039M4004V); dexamethasone sodium phosphate injection (Guizhou Tiandi Pharmaceutical Co., Ltd., Xingyi, Guizhou, China; specification: 1 mL:5 mg, batch number: H52020477); fetal bovine serum (FBS) and L-DMEM (Gibco, Grand Island, NY, USA); ELISA kit (NOVUS, Centennial, CO, USA; batch numbers: 890860, 546999, 101808); HE staining kit, Giemsa staining kit, and protein concentration detection kit (Beijing Solarbio Biotechnology Co., Ltd., Beijing, China; batch numbers: 20200629, G1020, 20200817); Trizol (THERMO, Milwaukee, WI, USA; batch number: 284912); RT-PCR kit (TAKARA, Kusatsu, Shiga, Japan; batch numbers: RR047A, RR820A); SDS-PAGE gel kit (Bio-Rad, Hercules, CA, USA; batch number: 64341430); Hoechst33258 staining solution (Beijing Solarbio Biotechnology Co., Ltd., Beijing, China; specification: 1 mg/mL, batch number: C0021); primary antibodies of *TLR4*, *NF-κB p65*, *MyD88*, and IκBα (batch numbers: Q9QUK6, Q04206, Q99836, P25963, Cell Signaling Technology, Beverly, MA, USA); rabbit anti-mouse phosphorylated antibodies against p-*NF-κB p65* and p-IκBα (Affinity, Cincinnati, OH, USA; batch numbers: AF2006, AF2002); and an ultrasensitive chemiluminescence kit (Affinity, Cincinnati, OH, USA; batch number: 1824a01).

### 2.2. Animals

Forty specific pathogen-free (SPF) Kunming (KM) mice of both sexes, weighing 25 ± 2 g, were offered by the Laboratory Animal Center of Guizhou Medical University (animal use license No. SCXK (Qian) 2018-0001; production license No. SCXY (Qian) 2018-0001). Animals were maintained at 25 ± 2 °C, with ambient humidity maintained at 50–70% with a 12 h photoperiod. All animals were acclimated for one week before any experimental procedures. The Animal Ethics Committee of Guizhou Medical University approved all animal protocols under approval No. 2101208.

### 2.3. Instruments

A biosafety cabinet, CO_2_ constant-temperature cell incubator, cryomicrotome, and MultiSkan3 microplate reader (Thermo Fisher, Waltham, MA, USA); a live-cell workstation (Olympus, Tokyo, Japan); a pathological tissue section scanner (Nano Zoomer, Hamamatsu City, Shizuoka, Japan); an automatic blood analyzer (Mindray, Shenzhen, Guangdong, China); a vortex oscillator (SCILOGEX, Rocky Hill, CT, USA); an electrophoresis instrument, gel imager, and RT-PCR system (Bio-Rad, Hercules, CA, USA); a light microscope (Nikon, Tokyo, Japan); an ultra-low temperature high-speed refrigerated centrifuge (Eppendorf, Hamburg, Germany); and an ultrapure water machine (Millipore, Billerica, MA, USA) were utilized in this experiment.

### 2.4. Isolation, Extraction, Culture and Identification of hUC-MSCs

Umbilical cords were obtained from healthy full-term neonates delivered by cesarean section, following written informed consent from the donors. Before collection, the mother’s good health status was confirmed, with no genetic diseases or infectious diseases in the family. Following collection, the umbilical vessels and outer membrane were carefully excised under aseptic conditions, and Wharton’s jelly was subsequently isolated. Wharton’s jelly was minced into approximately 1 mm^3^ fragments using sterile scissors and placed into 25 cm^2^ culture flasks for explant (adherent) culture. Low-glucose DMEM with 10% FBS was then added. Cultures were kept at 37 °C with 5% CO_2_ for primary expansion. The original medium was refreshed 3 days later, and cells migrated out of the tissue pieces at about 1 week after inoculation. Upon attaining 80–90% confluence, cells were dissociated by 0.25% EDTA–trypsin and subcultured. Cells at passage 5 were collected, and the protein levels of CD34, CD45, CD90, and CD105 (BD Biosciences, Mumbai, India) were assessed by flow cytometry using a Beckman FC500MCL system (Beckman Coulter, Miami, FL, USA). Additionally, hUC-MSCs were identified as previously described [7], with corresponding isotype-matched antibodies serving as negative controls. Well-grown cells at the 5th passage were used in this experiment. Specifically, hUC-MSCs at the 5th passage were harvested and cultured to about 70% confluence in a 75-cm^2^ flask. After removing the culture medium, cells were washed twice with normal saline and incubated with 2 mL Hoechst 33258 solution (Hoechst 33258: PBS = 1:1000) for 20–30 min at 37 °C in a 5% CO_2_ incubator. Successful fluorescent staining was observed under a live-cell workstation and photographed. After Hoechst 33258 staining, the supernatant was discarded, and L-DMEM medium containing 10% FBS was used for further cell culture. On the following day, cells were digested, centrifuged, and resuspended in phosphate-buffered saline (PBS) for in vivo cell transplantation in mice.

### 2.5. Model Construction and Group Treatment

KM mice (25 ± 2 g) were randomly allocated to four groups—control, LPS model, LPS + DEX (dexamethasone), and LPS + hUC-MSCs—with 10 mice per group. Anesthesia was performed via injection of 1% pentobarbital sodium of 0.1 mL/10 g intraperitoneally, following which mice were positioned supine. After disinfection of the neck region, a small midline incision (~0.3 cm) was made to expose the trachea via blunt dissection. LPS (5 mg/kg) was administered intratracheally, and the nasal openings were briefly occluded for 5–10 s to facilitate uniform pulmonary distribution. Successful model establishment was indicated by the presence of respiratory distress, agitation, and tachycardia in mice. The incision was subsequently closed in layers, and animals were returned to their home cages for recovery and monitored for 30 min until full recovery from anesthesia. One hour after modeling, mice in the hUC-MSC group received an intravenous injection via the tail vein of 2 × 10^6^ fluorescently labeled hUC-MSCs. In the DEX group, 3 mg/kg dexamethasone was given intraperitoneally daily for 3 consecutive days. Mice in all groups received an equal volume of saline following the same schedule.

### 2.6. Animal Sampling

Twenty-four hours after treatment, mice were anesthetized before blood samples were obtained by enucleation. Samples were kept at room temperature for 30 min prior to centrifugation to obtain serum, which was preserved at −80 °C for subsequent ELISA. After blood collection, the trachea was exposed via a neck incision, and 0.3 mL of pre-chilled PBS was delivered into the lungs through tracheal cannulation. The liquid was gently aspirated several times with a syringe at 1 min intervals, and the collection was repeated 3 times to obtain BALF. For mice without alveolar lavage, lung tissues were harvested after open thoracotomy under deep anesthesia. Lung tissue from each group was fixed at 4 °C in 4% paraformaldehyde (PFA) for histopathological analysis, part of which was kept under −80 °C for subsequent proteomic analysis and molecular experiments.

### 2.7. Measurement of Indicators

#### 2.7.1. Lung Function Test in Mice

On day 4 after cell transplantation or at 24 h after the completion of DEX injection, mouse lung function was tested with a FlexiVent pulmonary function tester (Montreal, Quebec City, QC, Canada) as described in the literature. Briefly, animals were anesthetized by injection of pentobarbital sodium intraperitoneally. After spontaneous breathing disappeared after deep anesthesia, endotracheal intubation was performed and connected to the FlexiVent pulmonary function tester. Mechanical ventilation was conducted under the conditions of 21% oxygen content, 150 breaths/min respiration rate, 10 mL tidal volume, and 2 cm H_2_O positive end-expiratory pressure (1 cm H_2_O = 0.098 kPa). When breathing was stable, a bronchial challenge test was performed with methacholine (MCh). The concentration of MCh was increased sequentially to 0, 1.5, 3, 6, 12, 25, 50 and 100 mg/L, with an interval of 2 min and a nebulization time of 20 s to induce bronchial smooth muscle spasm in mice. Meanwhile, airway resistance (Rrs), airway elastic resistance (Ers), dynamic compliance (Crs), and main airway resistance (P-3Rn) were detected.

#### 2.7.2. Lung Wet-to-Dry Weight Ratio (W/D)

The upper lobe of the left lung was dissected from each mouse, surface blood stains were rinsed with PBS, and residual liquid was blotted to measure the wet weight (W). Tissues were dried at 60 °C for 2 days(48 h) until constant weight was achieved.

#### 2.7.3. Inflammatory Cell Profiling in Mouse Balf by Giemsa Staining

BALF samples were centrifuged at 3000 rpm at 4 °C for 10 min. PBS was used to suspend cell pellets; 200 μL of cell suspension was used for differential counting of inflammatory cells using an automatic blood analyzer, and another 200 μL of the suspension was collected, fixed, and stained with Wright–Giemsa stain. Inflammatory cells were visualized using a light microscope.

#### 2.7.4. hUC-MSCs in Lung Tissue Tracked Using Frozen Sections

Lung tissue was cryosectioned at 8 μm thickness, and Hoechst 33258-positive cells were quantified.

#### 2.7.5. Pulmonary Histopathological Analysis

Lung tissues fixed in 4% PFA were paraffin-embedded and sectioned at 4 μm thickness for hematoxylin and eosin (H&E) staining. Histopathological evaluation was performed independently by two experienced pathologists who were blinded to the experimental group assignments. For each mouse, five randomly selected high-power fields (×400 magnification) were examined. Lung injury was assessed using a semi-quantitative scoring system based on four histopathological parameters: (i) alveolar edema, (ii) alveolar and interstitial inflammatory cell infiltration, (iii) alveolar wall thickening, and (iv) hemorrhage. Each parameter was scored on a scale of 0 to 3 as follows: 0, normal (no abnormality); 1, mild (less than 25% of the field affected); 2, moderate (25–50% of the field affected); and 3, severe (more than 50% of the field affected). The total lung injury score for each animal was calculated as the sum of the scores for the four parameters (range, 0–12). The final score for each group was expressed as the mean ± SD of the total scores from all animals in that group.

#### 2.7.6. RT-PCR Detection of mRNA Expression

Lung tissues kept in liquid nitrogen were used for total RNA extraction using Trizol, followed by reverse transcription to synthesize cDNA. cDNA was subjected to PCR amplification. Primers targeting mouse *TLR4*, *MyD88*, and *NF-κB p65* were designed based on sequences retrieved from the NCBI database. PCR was performed using a Thermal Cycler Dice Real-Time System. GAPDH served as an endogenous reference for normalization, and relative transcript levels were quantified by the 2^−ΔΔCt^ method. Primer sequences (Sangon Biotech, Shanghai, China) are shown in Table 1.

#### 2.7.7. Enzyme-Linked Immunosorbent Assay (ELISA)

Mouse serum samples stored at −80 °C were collected, and the absorbance values of IL-1β, IL-6, and TNF-α at 450 nm were quantified following the ELISA kit instructions. Cytokines were quantified by reference to standard curves.

#### 2.7.8. Frozen Sections

Lung tissue from the cell therapy group stored at −80 °C was collected and restored to room temperature, then placed on a tray. The tissue was gently spread flat with forceps, and embedding agent was added to the surface of the entire tissue block. The sample was then placed on a quick-frozen table pre-set at −20 °C for solidification, transferred to a section carrier, and cryosectioned at 8 μm thickness. Visualization and imagination were conducted using a live-cell workstation microscope.

#### 2.7.9. Expression of TLR4-MyD88-NF-κB Pathway-Related Proteins Was Detected by Western Blot

Lung tissues stored at −80 °C were collected for total protein extraction on ice. Protein abundance was quantified by the BCA assay. Proteins were resolved in 10% SDS-PAGE and electroblotted onto PVDF membranes at room temperature. Membranes were incubated in 5% non-fat milk for 90 min to block nonspecific binding, followed by 4 °C incubation with primary antibodies (1:1000) overnight. Secondary antibody (1:10,000) incubation was performed at room temperature for 2 h. An enhanced chemiluminescence (ECL) substrate was used, and band intensities were quantified using an imaging system.

### 2.8. Statistics

SPSS 22.0 software was used to perform statistical analyses. All data are expressed as mean ± standard deviation (SD). Differences among multiple groups were analyzed using one-way analysis of variance (ANOVA) in GraphPad Prism 8.0.2. A *p* value of less than 0.05 was defined as significant.

## 3. Results

### 3.1. Isolation and Characterization of hUC-MSCs

**Figure 1:**
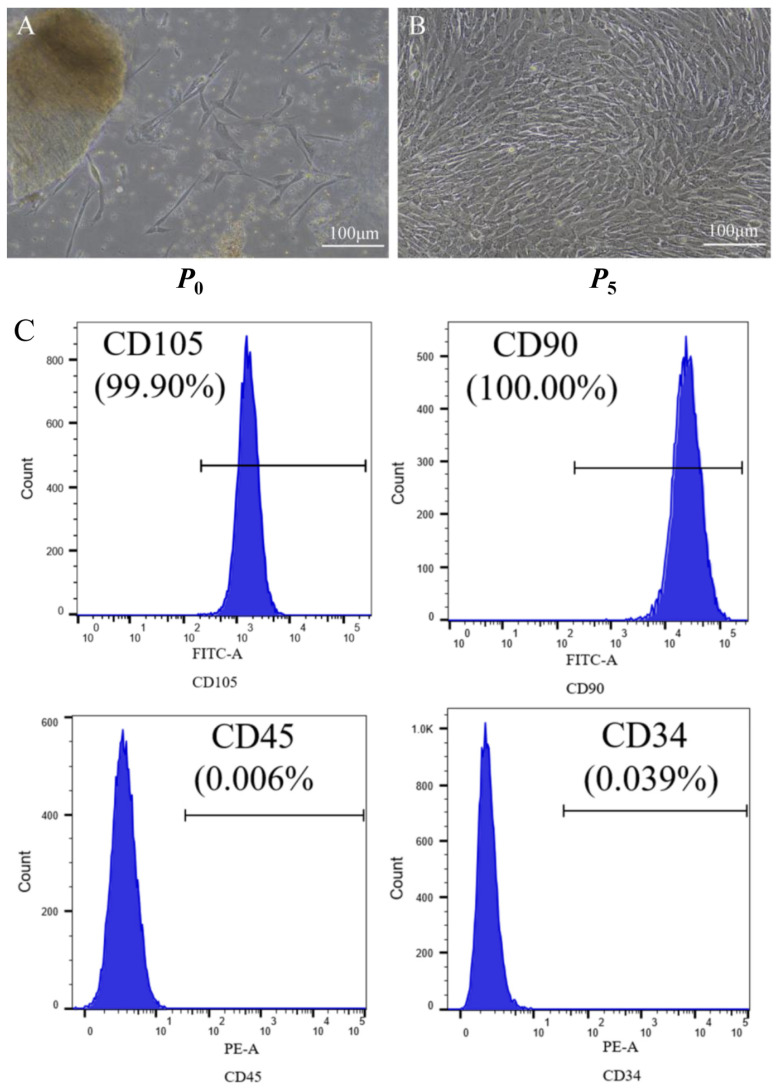
Morphology and flow cytometry characterization of hUC-MSCs. (**A**,**B**) Morphological characterization of hUC-MSCs: phase-contrast images of hUC-MSCs at initial seeding (*P*_0_) (**A**) and after the fifth passage (*P*_5_) (**B**). At *P*_0_, cells show initial adherence with low confluence. By *P*_5_, cells exhibit a typical spindle-shaped morphology and form a dense monolayer. Scale bars, 100 µm. (**C**) Flow cytometric characterization of passage-5 hUC-MSCs: the cells highly express MSC-specific markers CD105 (99.90%) and CD90 (100%), with minimal expression of hematopoietic markers CD45 (0.006%) and CD34 (0.039%), confirming a pure MSC population.

At one week post-seeding, cells began to migrate out of tissue explants and grew adherently, reaching approximately 50% confluence by day 14 (Figure 1A). After removing the tissue pieces and changing the medium, the cells grew rapidly, and the cell confluence reached over 80% after further culture for 3 to 5 days. Moreover, the cells proliferated more rapidly after passage and presented an elongated spindle or polygonal shape, forming a swirling or flowing appearance. The cell confluence reached 70–80% 3 to 5 days after each passage, at which point the cells were ready for the next passage (Figure 1B). hUC-MSCs were isolated by a mechanical method, and a large number of hUC-MSCs meeting the experimental needs were obtained by purification and expansion through culture and passage. Flow cytometry analysis showed that cells at the fifth passage expressed CD105^+^ (99.90%), CD90^+^ (100%), CD45^−^ (0.006%), and CD34^−^ (0.039%) (Figure 1C), which conformed to the biological characteristics of MSCs [7].

### 3.2. FlexiVent Lung Function Test Results

**Figure 2:**
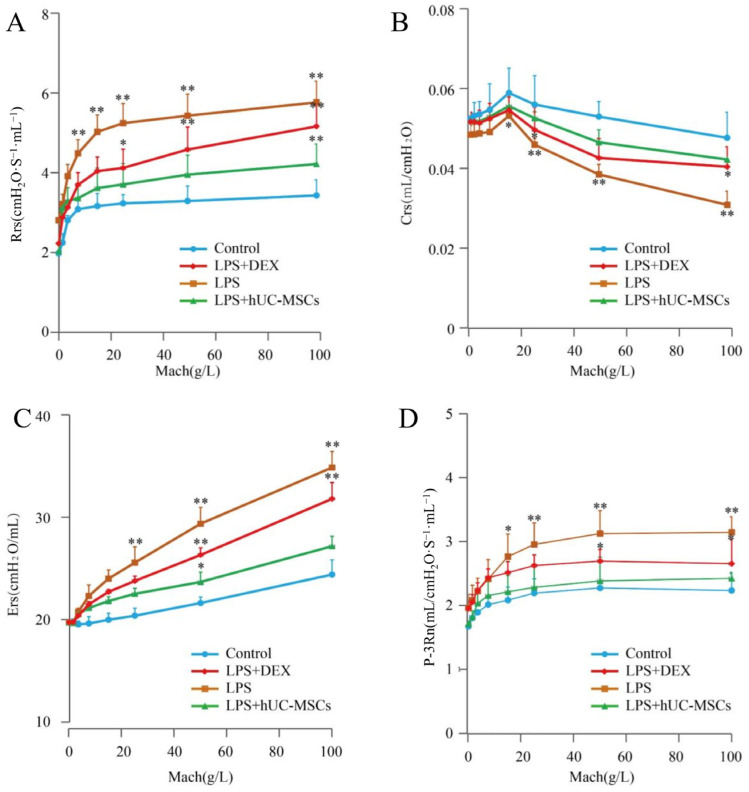
FlexiVent lung function test results. Pulmonary function parameters were evaluated after LPS-induced ALI and subsequent interventions. (**A**) Respiratory system resistance (Rrs), (**B**) dynamic compliance (Crs), (**C**) airway elastic resistance (Ers), and (**D**) central airway resistance (P-3Rn) were compared among control, LPS model, LPS + DEX, and LPS + hUC-MSC groups. LPS stimulation resulted in a significant upregulation in Rrs, Ers, and P-3Rn, along with a decrease in Crs (* *p* < 0.05, ** *p* < 0.01), reflecting marked impairment of lung mechanics. These abnormalities were significantly alleviated following treatment with dexamethasone or hUC-MSCs (* *p* < 0.05, ** *p* < 0.01).

LPS modeling significantly increased respiratory airway resistance (Rrs) (Figure 2A) while decreasing dynamic compliance (Crs) (Figure 2B), and also increased airway elastic resistance (Ers) (Figure 2C), and main airway resistance (P-3Rn) (Figure 2D) (*p* < 0.05, *p* < 0.01). Delivery of DEX or hUC-MSCs significantly reversed these alterations, restoring these parameters toward normal levels (Figure 2).

LPS modeling significantly increased respiratory airway resistance (Rrs) (Figure 2A), airway elastic resistance (Ers) (Figure 2B), and main airway resistance (P-3Rn) (Figure 2C), while decreasing dynamic compliance (Crs) (Figure 2D) (*p* < 0.05, *p* < 0.01). Delivery of DEX or hUC-MSCs significantly reversed these alterations, restoring these parameters toward normal levels (Figure 2).

### 3.3. hUC-MSC Functions in Lung W/D Ratio and Histopathology of ALI Model

**Figure 3:**
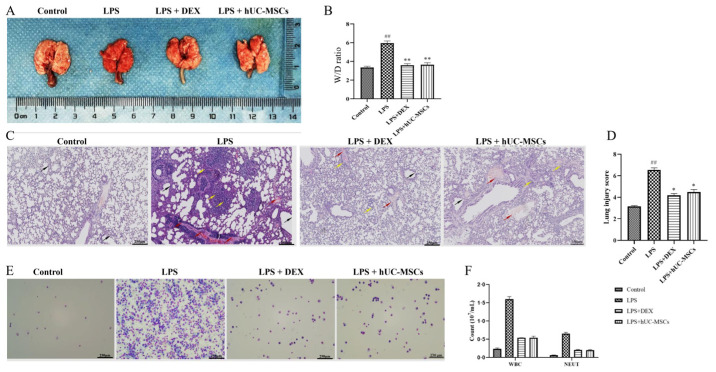
hUC-MSC-mediated amelioration of histopathological injury and inflammation in ALI mice. (**A**) Representative histological micrographs of lung tissues across groups. (**B**) Lung W/D ratio. (**C**) HE-stained lung sections showing histopathological changes (HE × 100, scale = 250 μm). Yellow, red, and black arrows indicate inflammatory cell infiltration, hyperemia, and alveolar walls, respectively. (**D**) Lung injury scores. (**E**) Inflammatory cell infiltration in BALF samples (HE × 100, scale bars, 250 µm or 100 µm, *n* = 8). (**F**) WBC and neutrophil (NEUT) count in BALF. ^##^ *p* < 0.01 vs. control; * *p* < 0.05, ** *p* < 0.01 vs. model group.

Mice in the model group showed a markedly increased lung W/D ratio accompanied by obvious pulmonary congestion and edema (Figure 3A,B) (*p* < 0.01), confirming successful induction of the ALI model. In the LPS + DEX and LPS + hUC-MSC groups, these pathological changes were progressively attenuated (Figure 3A,B). H&E staining demonstrated normal lung architecture in the control group, with no evident inflammatory infiltration or alveolar wall alterations. In contrast, the model group exhibited pronounced leukocyte infiltration, marked alveolar wall thickening, and severe alveolar edema, necrosis, and capillary hemorrhage (Figure 3C), resulting in significantly elevated histopathological scores (Figure 3D) (*p* < 0.01). Treatment with either DEX or hUC-MSCs markedly alleviated these pathological changes, including reduced alveolar wall thickness, edema, necrosis, hemorrhage, and leukocyte infiltration, with histological scores approaching those of the control group (Figure 3C,D). Notably, hUC-MSCs achieved a therapeutic effect comparable to that of DEX.

### 3.4. hUC-MSCs Alleviate Inflammatory Cell Accumulation in Mouse BALF

The model group showed markedly increased leukocyte and neutrophil counts in BALF (*p* < 0.01) relative to control (Figure 3E,F). Conversely, both LPS + DEX and LPS + hUC-MSC groups showed markedly reduced inflammatory cell numbers (*p* < 0.05) (Figure 3E,F), indicating attenuation of the inflammatory response in ALI mice.

### 3.5. Tracking the Colonization of hUC-MSCs in Lung Tissue Using Frozen Sections

**Figure 4:**
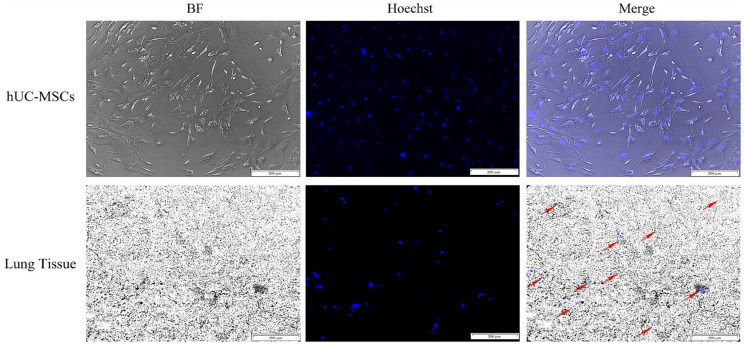
Colonization of hUC-MSCs in lung tissue. Representative bright-field (BF), Hoechst 33258 staining, and merged images showing the localization of hUC-MSCs in vitro and in tissue sections. The upper panel depicts cultured hUC-MSCs, while the lower panel shows lung sections from the hUC-MSC-treated group. Hoechst 33258-labeled hUC-MSCs are indicated by blue fluorescence, and their localization within peribronchial regions is highlighted with red arrow indicated in merged images. Scale bar, 200 µm.

Lung tissues from hUC-MSC-treated mice were sectioned at 8 μm to quantify Hoechst 33258-positive (blue fluorescent) cells. Most transplanted hUC-MSCs were detected in the peribronchial regions of lung tissue, suggesting their preferential homing to sites of injury. This distribution indicates that hUC-MSCs are capable of migrating toward and engrafting within inflamed lung areas, likely driven by inflammatory chemotactic signals (Figure 4).

### 3.6. Effects of hUC-MSCs on the TLR4, MyD88, and NF-κB mRNA Expression in Lung Tissue of ALI Mice

**Figure 5:**
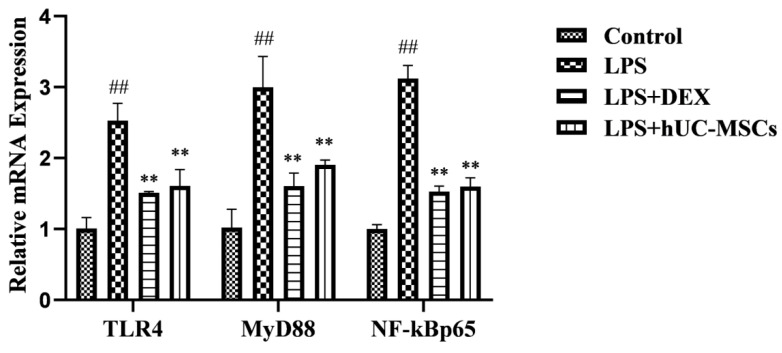
Impact of hUC-MSCs on the mRNA expression of *TLR4*, *MyD88* and *NF-κB* within pulmonary tissue in ALI model. Relative transcript levels of *TLR4*, *MyD88*, and *NF-κB p65* in lung tissues across groups. ^##^ *p* < 0.01 compared with controls; ** *p* < 0.01 compared with the LPS group.

LPS is known to induce an inflammatory cascade through upregulation of the TLR4/MyD88/NF-κB pathway. Transcriptional levels of *TLR4*, *MyD88*, and *NF-κB p65* were markedly elevated in the LPS group relative to controls. Conversely, the upregulated expressions were substantially reduced in both the LPS + hUC-MSC and LPS + DEX groups, close to control levels (*p* < 0.05). Our results revealed that hUC-MSCs can ameliorate lung injury by suppressing the TLR4/MyD88/NF-κB pathway (Figure 5).

### 3.7. hUC-MSCs Attenuate the Production of Inflammatory Mediators and Alleviate Lung Injury

**Figure 6:**
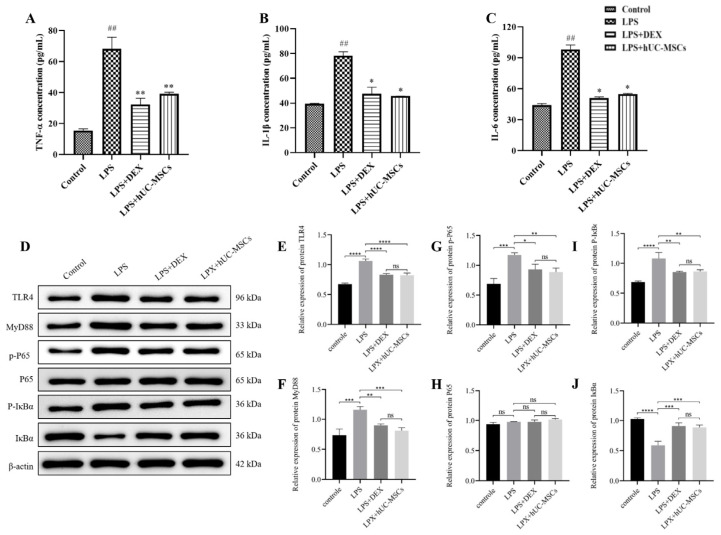
hUC-MSCs suppress inflammatory cytokines and TLR4/MyD88/NF-κB signaling in ALI mice. (**A**–**C**) Contents of TNF-α, IL-1β, and IL-6 in ALI mice following hUC-MSC treatment. (**D**) Western blot analysis of *TLR4*, *MyD88*, p-p65, p65, p-IκBα, and IκBα expression in lung tissues across experimental groups. (**E**–**J**) Densitometric quantification of the proteins shown in (**D**). ^##^ *p* < 0.01, vs. control group; * *p* < 0.05, ** *p* < 0.01, *** *p* < 0.001 **** *p* < 0.0001, vs. LPS group, ns means for not significant.

*TLR4* can recruit *MyD88*, leading to activation and nuclear translocation of NF-κB, which promotes the release of cytokines such as nitric oxide (NO) and IL-1β. In line with this pathway, levels of TNF-α, IL-1β, and IL-6 were substantially upregulated in the model group (*p* < 0.01) in serum (Figure 6A–C). Conversely, these cytokine levels were reduced to varying extents in both the hUC-MSC and LPS + DEX groups relative to the model group (Figure 6A–C). Our data revealed hUC-MSCs may alleviate lung injury in ALI by inhibiting cytokine production.

### 3.8. hUC-MSC-Mediated Suppression of TLR4/MyD88/NF-κB Pathway in ALI Lungs

Western blot analysis revealed that protein levels of *TLR4*, *MyD88*, p-p65, and p-IκBα were significantly elevated in the LPS group compared with controls (Figure 6D–J). In contrast, these proteins were markedly downregulated in both hUC-MSC and DEX groups (Figure 6D–J) (*p* < 0.05), with no significant differences between the two treatment groups (Figure 6).

## 4. Discussion

Acute lung injury (ALI) is a serious pulmonary disease marked by a high clinical mortality rate [1]. Currently, highly effective treatments with few adverse effects are still lacking [23]. Controlling excessive inflammation is considered critical in ALI therapy, because overproduction of pro-inflammatory cytokines increases capillary permeability and exacerbates tissue damages [6]. LPS not only activates *TLR4* on the surface of macrophages to trigger immune system activation, but also induces cytokine secretion by immune cells, which can even lead to septic shock [24,25,26,27]. The *MyD88*-dependent *TLR4* signaling pathway is a key upstream regulator of NF-κB activation. Under physiological conditions, NF-κB remains inactive in the cytoplasm as a component of an NF-κB/IκB complex. Under LPS challenge, IκB-α is phosphorylated and degraded, leading to the nuclear migration of NF-κB, thereafter promoting the pro-inflammatory mediators. This process amplifies inflammatory signaling and contributes to an excessive inflammatory cascade, often termed a cytokine storm, thereby exacerbating lung injury [28].

As adult stem cells, hUC-MSCs exhibit unique biological properties and are considered ideal seed cells for ALI cell therapy. MSC-based cell therapy has been actively investigated over the past few decades. To date, over 800 clinical trials investigating MSCs have been enrolled [29], including studies targeting Coronavirus Disease 2019 (COVID-19) (ClinicalTrials.gov, NCT numbers: NCT04269525, NCT04288102, NCT04313322 and NCT04273646) [30,31]. In addition, several clinical studies have reported beneficial efficacy of MSCs in LPS-induced ALI [32,33]. This study focused on the molecular mechanisms by which hUC-MSCs produce therapeutic benefits in ALI induced by LPS, suggesting that hUC-MSCs may ameliorate ALI via downregulating the TLR4–MyD88–NF-κB pathway. Our results demonstrate that intravenously administered hUC-MSCs localize to injured lung tissue, attenuate the inflammatory cascade, and thus improve pulmonary function in ALI mice.

hUC-MSCs have the following features that make them promising seed cells for ALI cell therapy [7]. First, hUC-MSCs exhibit low immunogenicity and immunosuppressive properties, with a wide range of immunomodulatory functions, which are conducive to both autologous and allogeneic transplantation [16,17]. Additionally, both exogenous and endogenous hUC-MSCs demonstrate a potent intrinsic tendency to migrate to damaged and inflamed tissues [34,35,36]. Accumulating studies have shown that various growth factors can induce the migration of hUC-MSCs to the inflammatory regions [37].

Second, hUC-MSC-derived soluble factors and microvesicles mediate paracrine actions that suppress inflammation and facilitate microenvironment remodeling and tissue regeneration [37,38]. Moreover, hUC-MSCs may differentiate into lung parenchymal cells, contributing to the replacement of injured cells and tissues, although this remains controversial. Finally, as a living biological drug, hUC-MSCs can exert their therapeutic effects for a certain period of time after transplantation into model animals, without causing severe adverse reactions in the body unlike glucocorticoids (e.g., DEX) [38,39]. Therefore, hUC-MSCs are capable of inhibiting the progression of ALI.

Targeting the TLR4/MyD88 pathway [40] or NF-κB activation [41,42] has been reported to attenuate LPS-induced ALI, underscoring the pivotal role of this signaling axis in disease progression. In this study, hUC-MSCs alleviated lung injury and reduced systemic and local inflammatory responses. To obtain better understanding of the precise mechanisms underlying this effect, we detected the role of hUC-MSCs in the TLR4/MyD88/NF-κB pathway. Our findings suggest hUC-MSCs suppress LPS-induced inflammatory responses in ALI by downregulating key mRNA and protein levels in lungs by means of the TLR4/MyD88/NF-κB pathway.

We acknowledge two major limitations. First, although our data demonstrate a strong association between hUC-MSC treatment and TLR4/MyD88/NF-κB suppression, causal evidence is lacking. Our current experimental design only detects endpoint mRNA and protein expression levels and cannot verify the temporal sequence of signaling events. However, the sequence TLR4 → MyD88 → NF-κB is a well-characterized pathway in the literature [19,23,25,26,27,28], and our findings of coordinated suppression across all components are consistent with this established framework. Future studies using pathway-specific agonists or antagonists are needed to confirm that this pathway is essential for the observed therapeutic effects.

Second, cytokines were measured in serum rather than BALF. Although serum cytokine levels reflect systemic inflammation, BALF analysis would provide more direct evidence of local pulmonary anti-inflammatory activity. Future studies will include BALF cytokine measurements to strengthen our conclusions.

## 5. Conclusions

To summarize, hUC-MSCs effectively ameliorate ALI, alleviate lung injury symptoms, and exert anti-inflammatory and pulmonary protection. The mechanism of action is linked to modulating the TLR4/MyD88/NF-κB pathway, inhibiting the generation of cytokines, and rescuing pulmonary inflammation. Our work clarifies the potential mechanism by which hUC-MSCs treat LPS-induced ALI, provides an objective experimental foundation, and offers theoretical support for further research on ALI treatment. Additionally, it presents a new perspective for MSC-based cell therapy in the clinical treatment of ALI.

## Figures and Tables

**Table 1:** Primer sequences.

Gene	Forward	Reverse	Length (bp)
*TLR*4	AGATCTGAGCTTCAACCCCTTG	AGTTTGAGAGGTGGTGTAAGCC	143
*MyD*88	AAGATGACCCTGGGAGCCCTA	CTCAGGCCAGTCATCATTGAACA	130
*NF-κB p*65	TCGAGTCTCCATGCAGCTACGG	CGGTGGCGATCATCTGTGTCTG	93
*GAPDH*	GGTTGTCTCCTGCGACTTCA	TGGTCCAGGGTTTCTTACTCC	183

## Data Availability

The raw data supporting the conclusions of this article will be made available by the authors on request.

## Abbreviations

ALI: acute lung injury; ANOVA: one-way analysis of variance; ARDS: acute respiratory distress syndrome; BALF: bronchoalveolar lavage fluid; BCA: bicinchoninic acid; BM-MSCs: bone marrow-derived mesenchymal stem cells; BSL-2: Biosafety Level 2; CD: Cluster of Differentiation; COVID-19: Coronavirus Disease 2019; CRO: Contract Research Organization; Crs: dynamic compliance; DEX: dexamethasone; ECL: enhanced chemiluminescence; ELISA: Enzyme-Linked Immunosorbent Assay; Ers: airway elastic resistance; FBS: fetal bovine serum; GLP: Good Laboratory Practice; HE: hematoxylin and eosin; hUC-MSCs: human umbilical cord-derived mesenchymal stem cells; IκBα: inhibitor of κB α; IL-1β: interleukin-1β; IL-6: interleukin-6; IL-10: interleukin-10; KM: Kunming; L-DMEM: low-sugar Dulbecco’s Modified Eagle Medium; LPS: lipopolysaccharide; MCh: methacholine; MSCs: mesenchymal stem cells; MyD88: myeloid differentiation factor 88; NCT: National Clinical Trial; NF-κB: nuclear factor-κB; NF-κB p65: nuclear factor-κB p65; NEUT: neutrophil; NO: nitric oxide; *P*_0_: passage 0; *P*_5_: passage 5; PBS: phosphate-buffered saline; PEEP: positive end-expiratory pressure; p-IκBα: phosphorylated IκBα; p-p65/p-NF-κB p65: phosphorylated NF-κB p65; P-3Rn: main airway resistance; PVDF: Polyvinylidene Difluoride; Rrs: respiratory system resistance; RT-PCR: Real-Time Polymerase Chain Reaction; SDS-PAGE: Sodium Dodecyl Sulfate–Polyacrylamide Gel Electrophoresis; SD: standard deviation; SPF: specific pathogen-free; TLR4: Toll-like receptor 4; TNF-α: tumor necrosis factor-alpha; WBC: white blood cell; W/D: wet-to-dry.
